# High-Temperature Performance Enhancement of Asphalt Binders Modified with Single-Use Masks: A Rheological Analysis with Predictive Modeling

**DOI:** 10.3390/polym17131746

**Published:** 2025-06-24

**Authors:** Alaaeldin A. A. Abdelmagid, Guanghui Jin, Guocan Chen, Baotao Huang, Yiming Li, Aboubaker I. B. Idriss

**Affiliations:** 1 School of Civil Engineering, Quanzhou University of Information Engineering, Quanzhou 362000, China; alaaeldin@qzuie.edu.cn (A.A.A.A.); cgc@qzuie.edu.cn (G.C.); huangbaotao2024@qzuie.edu.cn (B.H.); 2 Engineering Research Center of Green Building and Environmentally Friendly Materials, Fujian Universities, Fuzhou 350116, China; 3 School of Civil and Transportation Engineering, Northeast Forestry University, Harbin 150040, China; jin1650@nefu.edu.cn; 4 College of Mechanical and Electrical Engineering, Northeast Forestry University, Harbin 150040, China; 5 Department of Mechanical Engineering, Faculty of Engineering Science, University of Nyala, Nyala P.O. Box 155, Sudan

**Keywords:** single-use mask, rutting resistance, high-temperature performance, rheological properties, modified asphalt, response surface methodology

## Abstract

Due to high temperatures and repeated load, asphalt pavements commonly experience rutting distress, a challenge that can be considerably reduced by modifying the binder components. This research focused on evaluating the performance of asphalt binders with single-use masks (SUMs) when subjected to high temperatures. For this purpose, dynamic shear rheometer (DSR)-based frequency sweep, temperature sweep, and multiple stress creep recovery (MSCR) experiments were performed on various asphalt binders, including both unmodified and SUM-modified (SUMM) samples. To explore the effects of temperature, SUM content, and loading frequency on the rutting performance of the SUMM samples, a statistical modeling-based response surface methodology (RSM) was utilized, enabling the creation of predictive mathematical models. To investigate the internal morphology of the binders, fluorescence microscopy (FM) was applied. Data from rheological analyses revealed that the addition of SUM markedly boosted the high-temperature resistance of the asphalt binder. Findings from the MSCR analysis indicated that the SUMM samples achieved lower nonrecoverable compliance (J_nr_) and greater percent recovery (R) values than the reference asphalt, suggesting that SUMs significantly enhance the binder’s resistance to rutting. Data analysis demonstrated that the chosen independent variables had a considerable effect on the asphalt’s complex modulus (G*) and rutting performance (G*/sin (δ)), prompting the formulation of predictive models for rutting susceptibility. Moreover, the FM examination demonstrated that the SUM was homogeneously integrated across the asphalt matrix.

## 1. Introduction

Since the onset of the COVID-19 pandemic, mask-wearing has evolved from a temporary precaution to a deeply ingrained daily routine. This transformation can be ascribed to a number of factors, including government mandates, individual risk assessments, and widespread media influence [1,2]. Initially introduced as an essential public health measure, mask-wearing has continued even as the immediate threat of the virus has subsided, reflecting lasting shifts in social behavior [3]. The repeated use of masks, reinforced by the perceived sense of safety and the desire to avoid potential health risks, has made mask-wearing an automatic practice [4]. However, this widespread adoption has led to significant environmental and economic concerns—specifically, the disposal of masks. The improper disposal of masks creates major waste management challenges, impacting both the environment and the economy at large [5,6,7], and it can result in their accumulation in rivers and oceans, posing a substantial threat to marine life [8]. Recent estimates indicate that, annually, between 0.15 and 0.39 million tons of mask waste are discarded into the ocean, jeopardizing marine ecosystems [9]. Disposing of masks through conventional methods—such as high-temperature incineration—results in a dual environmental impact. Not only does it exacerbate global warming by emitting greenhouse gases, but it also releases harmful toxins into the air, further polluting the environment [10]. Discarding used masks in landfills is not a viable solution, as it can contaminate the soil [11,12]. Additionally, masks take centuries to decompose, leading to long-term environmental issues [13]. To address this problem, one promising solution involves integrating mask waste into asphalt, which could pave the way for more sustainable road construction.

Single-use masks (SUMs) are composed of multiple polymer materials, comprising polycarbonate, polypropylene (PP), and polyester [14]. Of these, polypropylene (PP) is the primary constituent [15]. Its large molecular weight makes it suitable for use in asphalt and asphalt mixtures. Studies have demonstrated that PP-modified asphalt exhibits superior properties compared to its unmodified counterpart [16,17]. Furthermore, incorporating PP can enhance the deformation resistance, viscosity, and thermal stability of asphalt [18,19]. Chen et al. created a modified asphalt formulation incorporating waste organic rectorite (OR) and PP. This modified asphalt, which included 1.5% OR and 4% PP, exhibited improved plasticity and ductility [20]. Moreover, the inclusion of PP contributed to increased deformation resistance, Marshall stability, and indirect tensile strength in asphalt mixtures [21,22]. When compared to the control mixture, the PP-modified asphalt mixture showed respective increases of 67.5%, 38%, and 26.3% in porosity, flow value, and Marshall stability [22]. Wang et al. observed that asphalt mixtures reinforced with PP fibers achieved superior crack resistance and greater tensile strength in comparison to mixtures containing polyester and different polymeric fibers [23]. One more study demonstrated that adding both SBR and PP to asphalt significantly improved wear resistance and reduced permanent deformation [24]. Furthermore, research has shown that PP-modified asphalt can alleviate wear-induced pavement damage [25]. In addition, PP materials have been employed to lower pavement surface temperatures during the summer [26].

Rutting has emerged as a primary form of damage to asphalt pavements. At present, asphalt surfaces are prone to permanent deformation due to the combined impact of load and elevated temperatures [27]. Additionally, if maintenance is delayed, deformation will progressively worsen, leading to damage to the pavement structure. As a result, the road’s performance declines, which accelerates the deterioration of the asphalt pavement’s service life. This situation is detrimental to both economic and environmental outcomes [28]. At intermediate and elevated temperatures, the Superior Performing Asphalt Pavements (Superpave) framework uses a dynamic shear rheometer (DSR) to measure the elasticity and stiffness of asphalt binders, which helps in evaluating their resistance to rutting. Temperature and frequency sweep (FS) tests are commonly used to analyze how asphalt binders respond to varying loading frequencies and temperatures by estimating their viscoelastic characteristics, such as the complex modulus (G*) and phase angle (δ) [29,30,31,32]. The rutting indicator (G*/sin (δ)) was established to evaluate rutting resistance. G*/sin (δ) combines G*—a measure of total binder stiffness—with δ, which reflects the viscoelastic balance between elastic and viscous behavior. Higher values indicate greater elasticity and stiffness, which are critical for high-temperature performance. Numerous laboratory studies have utilized this parameter to examine the rutting susceptibility of both conventional and modified binders, employing the DSR to replicate field conditions [33,34,35,36]. The multiple stress creep recovery (MSCR) test was introduced as an enhancement to G*/sin (δ) to more accurately assess the susceptibility of asphalt binders to rutting at elevated temperatures [37]. This technique implements nonrecoverable compliance (J_nr_) as a new indicator that more precisely gauges rutting propensity in modified asphalt binders than the traditional parameter G*/sin (δ). Additionally, the MSCR test allows for the measurement of the binder’s recovery. A wide range of experimental and field research has provided evidence supporting the applicability of this approach to conventional and modified bituminous materials [38,39,40,41].

The response surface methodology (RSM) is a statistical and mathematical approach used to develop models, design experiments, assess the effects of various factors, and optimize processes [42,43,44,45,46]. To further understand the performance characteristics of asphalt pavements, there is growing interest in the use of the RSM to explore how different variables affect performance [47,48,49,50,51]. Frequency and temperature are among the primary determinants influencing rutting deformation in asphalt pavements [52]. The RSM provides an efficient means to analyze how these variables interactively influence the deformation behavior of the asphalt binder.

Drawing from the existing literature, this study is motivated by the pressing need to address both the declining performance of traditional asphalt and the environmental concerns brought on by inappropriate SUM disposal. Despite the promising benefits of using waste-derived modifiers, a gap remains in understanding the incorporation of SUMs as a modifier for asphalt binders. Furthermore, the extent to which both temperature and frequency influence the rutting tendency of asphalt binder with SUM modification remains insufficiently understood. Consequently, this research intends to thoroughly scrutinize the impact of different concentrations of SUMs on the high-temperature performance of asphalt binders through laboratory experiments. Additionally, a statistical approach (RSM) was used to investigate the impact of temperature, SUM dosages, and loading frequency on the rutting behavior of binders. While the peak accumulation of SUM waste was driven by the COVID-19 pandemic, their persistence in landfills and natural environments necessitates sustainable repurposing strategies. This study focuses on recycling existing SUM waste rather than promoting new mask production, aligning with circular economy principles. The main goals are outlined as follows:

- Investigating the impact of SUM incorporation into asphalt binders on their viscoelastic behavior and G*/sin (δ) through assessments involving temperature variations and FS analyses;
- Utilizing the RSM to explore the combined influences of SUM concentration, temperature, and loading frequency on binder performance indicators, specifically G*/sin (δ) and G*, and subsequently formulating predictive models for these characteristics;
- Evaluating the performance of SUM-modified asphalt binders (SUMMs) at elevated temperatures by determining percent recovery (R) and J_nr_, using MSCR testing;
- Employing fluorescence microscopy (FM) to examine the surface morphology of the asphalt binders.

## 2. Materials and Test Program

### 2.1. Raw Materials

AH-70# asphalt binder—supplied by China Petroleum and Chemical Corporation in Beijing—was selected as the base binder, with its physical characteristics presented in Table 1. These properties were determined in accordance with the penetration grade AH-70# requirements specified in the Chinese Standard JTG F40-2004 [53], Technical Specifications for Construction of Highway Asphalt Pavement.

**Figure 1:**
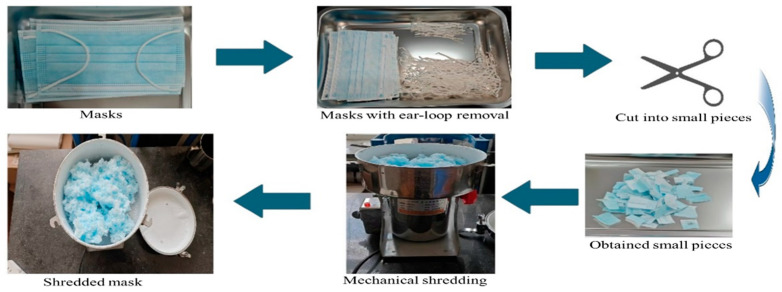
SUM preparation.

Adhering to laboratory protocols and governmental regulations, unused SUMs were selected for testing, and their physical characteristics are reported in Table 2. Prior to use, the metal components and cotton threads of the SUMs were removed. The SUMs were then cut into pieces approximately 50 mm in length and 30 mm in width. These SUM fragments were subsequently placed into a crusher to obtain shredded SUMs, as depicted in Figure 1.

**Figure 2:**
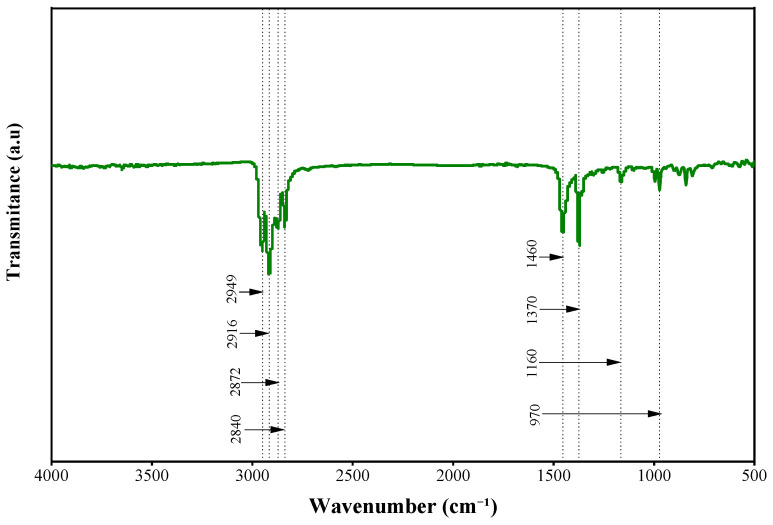
FTIR spectra of SUMs.

Figure 2 displays the Fourier Transform Infrared (FTIR) test outcomes of the SUMs. The spectral data were collected over the range of 4000–500 cm^−1^ (wave number). FTIR analysis was carried out utilizing a Thermo Scientific Nicolet iS5 Spectrometer (Thermo Fisher Scientific, Waltham, MA, USA). The FTIR spectra of the SUMs exhibited peaks at 2950 cm^−1^, 2916 cm^−1^, 2872 cm^−1^, 2840 cm^−1^, 1460 cm^−1^, 1370 cm^−1^, 1160 cm^−1^, and 970 cm^−1^. An analysis of the SUMs‘ infrared spectrum against the standard polypropylene spectrum indicated that the material is composed of PP [58].

### 2.2. Preparation of SUMM Samples

**Figure 3:**
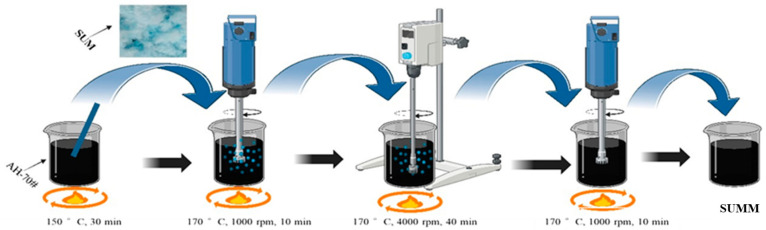
Preparation of SUMM samples.

All specimens were fabricated using both high-shear and mechanical mixers. Within this study, five different SUM concentrations were applied, specifically 2%, 4%, 6%, 8%, and 10% by weight relative to the asphalt. The preparation process began by heating 500 g of the base asphalt until it reached a molten condition, after which incremental quantities of the SUMs were gradually incorporated and then rotated at 1000 rpm within the mechanical mixer for a duration of 10 min. Following this, the blend underwent high-speed mixing at 170 °C and 4000 rpm for a duration of 40 min. To eliminate any residual air, the prepared blend was gently stirred at a low speed within a mechanical mixer for ten minutes, as illustrated in Figure 3.

### 2.3. Temperature Sweep Test

A temperature sweep test was performed to determine the rheological properties of the asphalt binder under high temperature conditions. A DSR was employed for this purpose (MCR301, Anton Paar, Graz, Austria). The test was carried out over a temperature range of 52–82 °C, with increments of 6 °C, under controlled strain conditions with 1.59 Hz frequency. A parallel plate configuration with a 0.001 m gap and a 0.025 m diameter was used for the test. The measurements were performed on both unaged samples and those aged utilizing the rolling thin-film oven (RTFO) procedure. The RTFO test, performed in accordance with ASTM D2872 [59], was used to simulate short-term aging of the samples. Time, temperature, and airflow parameters were rigorously controlled, with the duration set to 85 min, the temperature held at 163 °C, and the airflow rate regulated at 4000 ± 200 mL/min.

### 2.4. FS Test

To scrutinize the viscoelastic behavior of the SUMMs, FS experiments were conducted. The test was performed over a frequency range of 0.1 to 100 Hz under controlled strain at various temperatures from 40 °C to 80 °C, with 10 °C intervals. Parallel plates with a 0.001 m gap and a 0.025 m diameter were used.

### 2.5. MSCR Test

The MSCR test employs the principles of creep and recovery testing to evaluate the rutting resistance of asphalt binders. Recent research has demonstrated that the MSCR test produces outcomes that exhibit a closer association with rutting behavior in comparison with the high-temperature rheological factor G*/sin (δ). Consequently, the MSCR methodology, implemented in accordance with ASTM D7405-15 [60], was utilized to evaluate the rutting susceptibility of the SUMMs.

**Figure 4:**
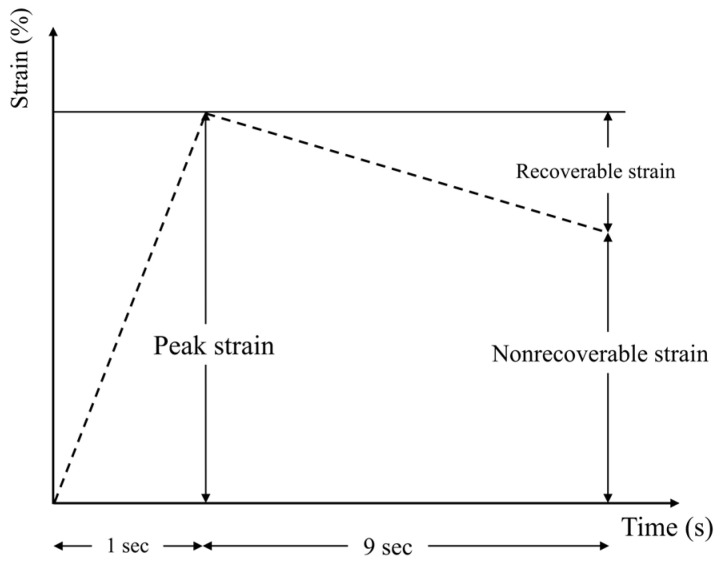
A representative creep–recovery cycle.

In this research, the MSCR test was carried out at 58 °C and 64 °C. Prior to testing, all asphalt binder samples underwent aging through the RTFO procedure. The DSR was employed to execute the test. The parallel plate configuration had a diameter of 0.025 m and a gap setting of 0.001 m. In the first 10 cycles, a constant shear creep stress of 0.1 kPa was utilized, comprising 1 s of loading followed by 9 s of recovery. Subsequently, a higher shear creep stress of 3.2 kPa was imposed for an additional 10 cycles. A representative cycle of the creep–recovery process is illustrated in Figure 4.

For every cycle, the values of two parameters, J_nr_ and R, are determined through the application of Equations (1) and (2).

(1) $$J_{nr}=\frac{Nonrecoverable\;starin}{Applied\;shear\;stress}$$

(2) $$R=\frac{Recoverable\;shear\;starin}{Peak\;strain}$$

### 2.6. RSM

The RSM is an experimental design approach employed to identify relationships between multiple independent variables and one or several response variables through mathematical modeling [61,62]. Furthermore, it serves to optimize the response outcome by adjusting the levels of the experimental factors. The effects of temperature, SUM dosage, and frequency as independent factors on the parameters G*/sin (δ) and G* were examined in this work using the RSM. Based on the Central Composite Design (CCD) technique, the experimental arrangement—along with the subsequent statistical assessment and construction of a response surface model—was undertaken through the use of Design-Expert software version 13. CCD is often favored because it is well suited for sequential experimental procedures and excels at producing accurate predictions throughout the entire design domain [63,64]. At the outset of the research, the following three independent factors were established: temperature (referred to as F_1_), frequency (referred to as F_2_), and SUM content (referred to as F_3_). The RSM suggested temperature settings of 40 °C, 60 °C, and 80 °C; frequency values of 0.1, 50.50, and 100 Hz; as well as SUM concentrations of 2%, 6%, and 10%. Table 3 presents the results derived from the variables identified through RSM analysis. A total of 19 experimental groups were established utilizing Design-Expert version 13.0 software, with 5 of these specifically assigned as the center points within the response surface design framework. These particular groups maintained constant levels of temperature, load frequency, and SUM content to facilitate the assessment of experimental error. The association between the independent variables and the response was investigated through the analysis of the 14 remaining experimental groups. Among the various modeling approaches considered, this study employed the quadratic polynomial regression model for predicting response values, owing to its demonstrated superior accuracy. The corresponding mathematical formulation is presented in Equation (3) [65]:

(3) $$Y=\beta_{0}+\sum_{i=1}^{n}\beta_{i}x_{i}+\sum_{i=1}^{n}\beta_{ii}x_{i}^{2}+\sum_{i<1}^{n}\beta_{ij}x_{i}x_{j}+\epsilon$$

Representing the intercept, linear, quadratic, and interaction coefficients, β_ij_, β_ii_, β_i_, and β_0_ respectively, these parameters define the model. Y stands for the predicted outcome, n is the count of factors considered, and ϵ denotes the random error. The independent variables are labeled as x_i_ and x_j_.

To evaluate model suitability and assess the influence of temperature, load frequency, SUM content, and their combined effects with G* and G*/sin (δ), ANOVA was applied.

### 2.7. Fluorescence Microscopy (FM)

**Figure 5:**
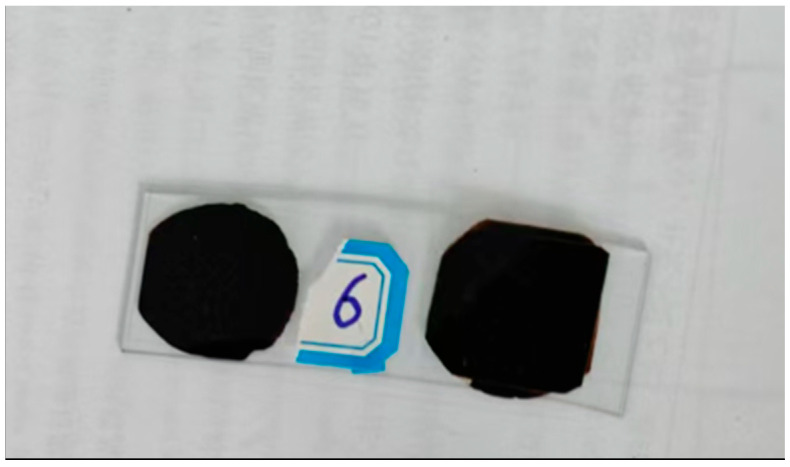
Sample for microscope.

Fluorescence imaging was applied to examine the distribution of the SUMs within the asphalt. To prepare the sample slides, a small droplet of the heated material was deposited onto a bottom glass slide and then covered with a top slide. This droplet, enclosed between the two slides, was gently compressed to create a thin uniform film. The sample (Figure 5) was subsequently observed at room temperature using Axio Observer 3 optical microscope (Carl Zeiss IMT Co., Ltd., Pudong, Shanghai, China).

## 3. Results and Discussion

### 3.1. Temperature Sweep Test

**Figure 6:**
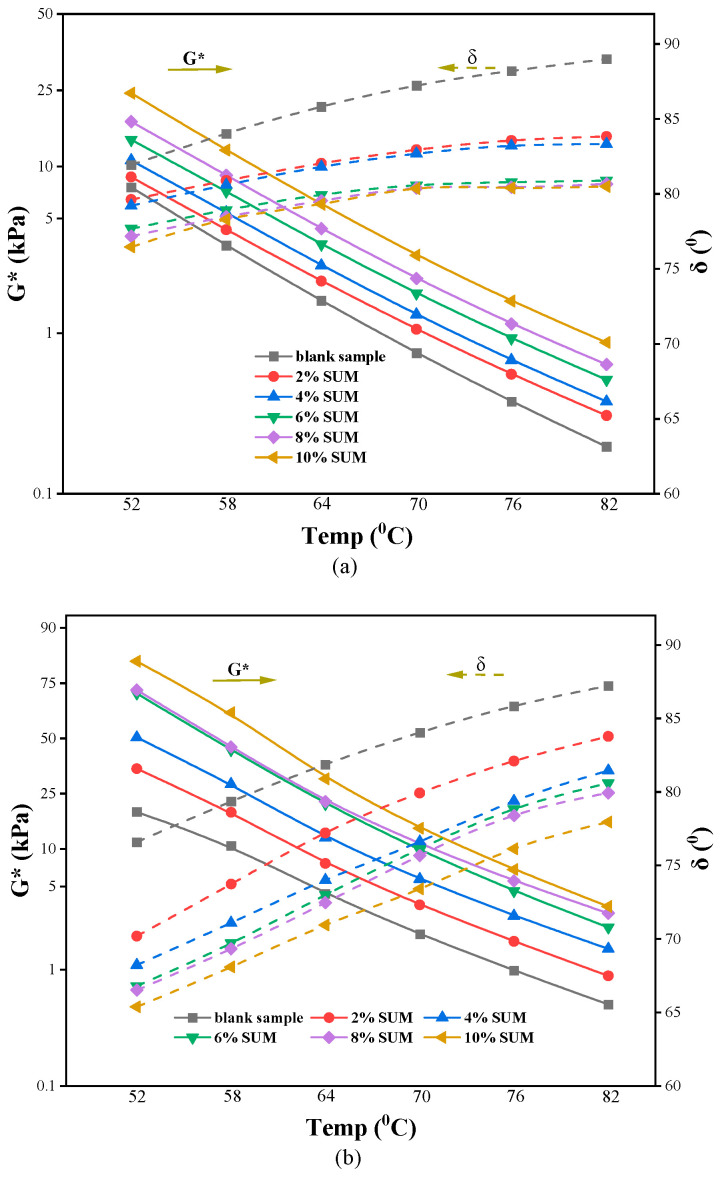
Impact of SUM dosage on G* and δ parameters measured (**a**) before and (**b**) after RTFO aging.

**Figure 7:**
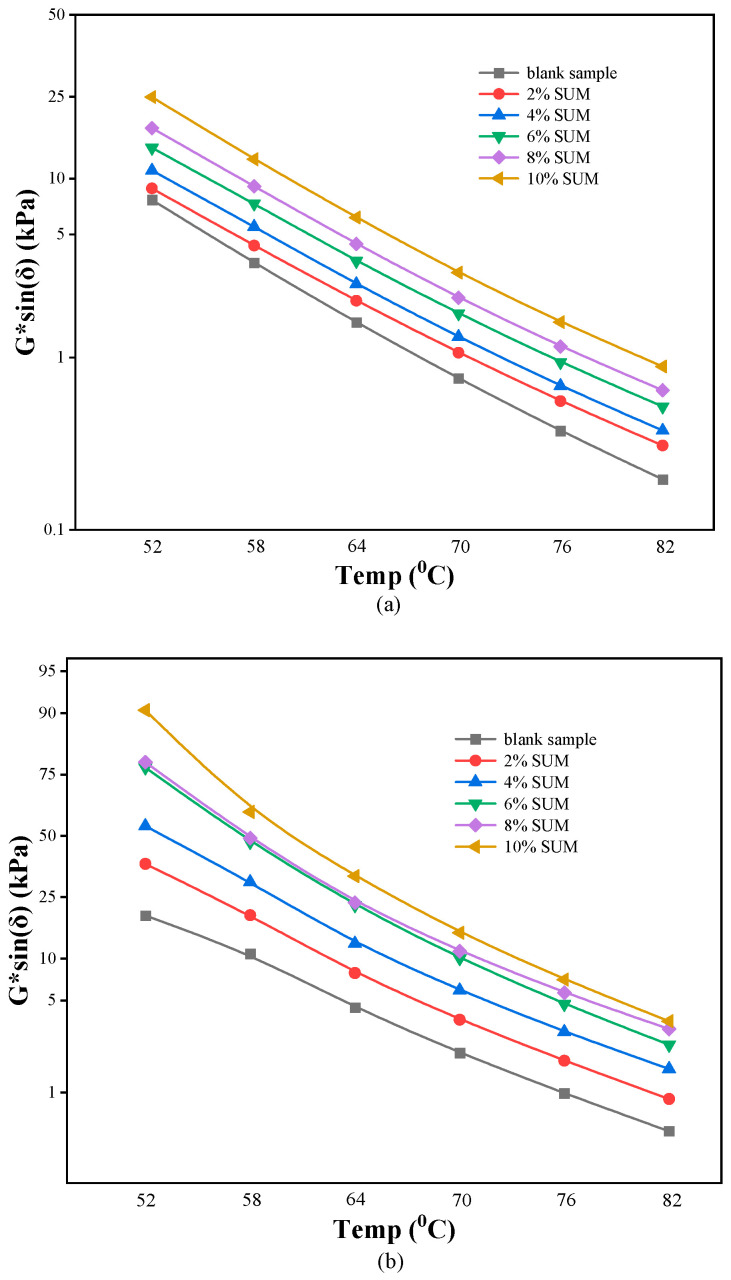
Impact of SUM dosage on G*/sin (δ) parameters measured (**a**) before and (**b**) after RTFO aging.

Figure 6 displays the G* and δ values for both the unmodified and SUMM samples, measured prior to and following RTFO aging. Figure 6a shows that increasing temperature results in a notable decrease in G*, which stems from the temperature-dependent flow behavior of asphalt and consequently influences its effectiveness in resisting rutting. Figure 6a demonstrates that the incorporation of SUMs significantly increases G* values, with these enhancements evident across a broad spectrum of temperatures. The observed improvement in G* values indicates that incorporating SUMs enhances the material’s ability to resist deformation under dynamic loading conditions. Relative to SUMMs and tested across different temperatures, the unmodified sample exhibited the lowest G* values and correspondingly reduced stiffness. Moreover, Figure 7a reveals that δ progressively declines as the concentration of SUM increases. As the test temperature increases, an upward trend in δ is observed, causing elasticity to decrease and viscosity to increase. Hence, the binder shifts toward a more viscous behavior. A steeper δ slope signifies that the asphalt is characterized by greater viscous behavior relative to elasticity, recovering its original shape through the process of energy dissipation. Moreover, under elevated temperatures, an increased δ value is unfavorable because it contributes to greater permanent deformation. Conversely, a lower δ under these conditions signifies a stronger elastic response from the asphalt binder, thereby improving its resistance to permanent deformation. Nevertheless, as the proportion of SUMs rises considerably, the δ values remain low even under elevated temperature conditions, suggesting a shift in the asphalt’s behavior from viscous to elastic. Furthermore, an increase in SUM concentration corresponds with a decrease in δ values, a trend that remains uniform regardless of the specific SUM content. This reflects the strong recovery capability exhibited by SUMMs. Results presented in Figure 6a reveal that asphalt binders modified with SUMs demonstrate superior elastic properties at high temperatures compared to the unmodified sample, potentially leading to improved mechanical performance of modified HMA when subjected to load.

According to the data presented in Figure 6b, G* and δ exhibit contrasting trends following the aging process when assessed across various temperatures. Data presented in Figure 7b indicate that incorporating SUMs leads to an increase in G* values, whereas δ values decline with higher SUM concentrations. An increase in G* parameters indicate stiffening behavior, while a decrease in δ values signifies greater elasticity. Subsequent to RTFO aging, the asphalt binder exhibits increased hardness alongside improved performance under high temperature conditions. During the aging process of asphalt, there is an increase in the volatile fractions of the light components, accompanied by a reduction in the viscous fractions of the related materials. Thus, the loss rate of asphalt’s viscous components slows down post-aging. Analysis of G* and δ data collected after aging reveals that asphalt binders formulated with higher SUM dosages undergo greater modifications at the same temperature than those prepared with lower dosages. As an illustration, at 64 °C, G* of the unmodified sample increased by 76.2%, 179.89%, 389.45%, and 407% following the addition of 2%, 4%, 6%, and 8% SUM content, respectively. Conversely, the δ of the same sample at this temperature decreased by 5.7%, 9.6%, 10.8%, and 11.5% when subjected to the same respective SUM concentrations.

**Figure 8:**
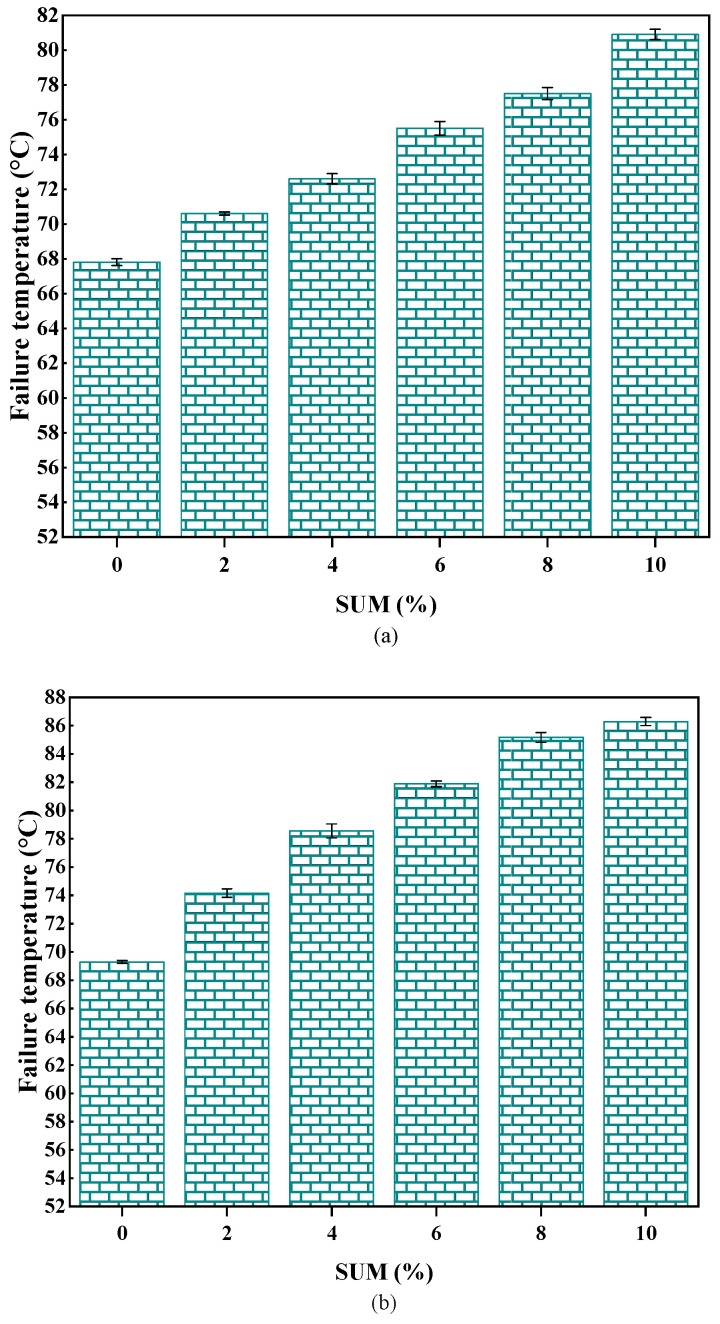
Impact of SUM dosage on failure temperature measured (**a**) before and (**b**) after RTFO aging.

Under high temperature conditions, G*/sin (δ) serves as a metric to assess the permanent deformation resistance of asphalt binders. According to established standards, G*/sin (δ) must be at least 1.0 kPa before RTFO aging and increase to a minimum of 2.2 kPa following RTFO aging. The G*/sin (δ) measurements of the unmodified sample, alongside SUMMs measured at different temperatures prior to RTFO aging, are revealed in Figure 7a. Figure 8a illustrates a noticeable decrease in G*/sin (δ) values corresponding to rising temperatures. As temperatures rise, the internal bonding of the asphalt weakens and softens, resulting in decreased resistance to rutting. The inclusion of SUMs enhances G*/sin (δ) under consistent temperature conditions. The observed improvement in the G*/sin (δ) parameter caused by the inclusion of SUM demonstrates that the additive strengthens asphalt’s ability to resist rutting under increased temperature conditions. According to Figure 7a, G*/sin (δ) for the unmodified specimen at 70 °C was recorded as 0.76 kPa, falling below the standard threshold of 1.0 kPa. However, when the SUM content reached 2% or higher, the value surpassed 1.0 kPa, indicating an improvement of one grade in the elevated temperature rating to 70 °C. This confirms that SUM effectively boosts the performance of asphalt binders at higher temperatures. This effect is attributed to the formation of a reinforcing polymer network created by the dispersed SUM particles within the asphalt matrix, which enhances the binder’s elastic properties and stiffness [66]. This three-dimensional network structure physically restricts the movement of asphalt molecules, leading directly to the observed increase in G and decrease in δ [66,67,68,69], a mechanism that will be confirmed by fluorescence microscopy in Section 3.5.

RTFO aging refers to a treatment where the asphalt binder undergoes oxidative reactions. Upon completion of RTFO aging, a significant increase in G*/sin (δ) was observed across all asphalt binders containing varying amounts of SUM when tested at a consistent temperature, as depicted in Figure 7b. For example, when 2%, 6%, and 10% SUM content were incorporated, the G*/sin (δ) values of the unmodified specimen at 64 °C showed increases of 78.85%, 406.68%, and 640.17%, respectively. This effect is explained by RTFO aging, which causes the asphalt to harden and reduces the quantity of SUM components it contains. Furthermore, the G*/sin (δ) values obtained from unaged asphalt binders were consistently lower than those measured in aged samples. SUMMs containing elevated levels of SUMs showed enhanced stability in their G*/sin (δ) parameters throughout the aging process, which could diminish oxidative degradation during RTFO aging and possibly result in aged asphalt that is less stiff. When contrasted with the unmodified asphalt, SUMM maintained the greatest G*/sin (δ) throughout the entire temperature range regardless of aging conditions.

**Figure 9:**
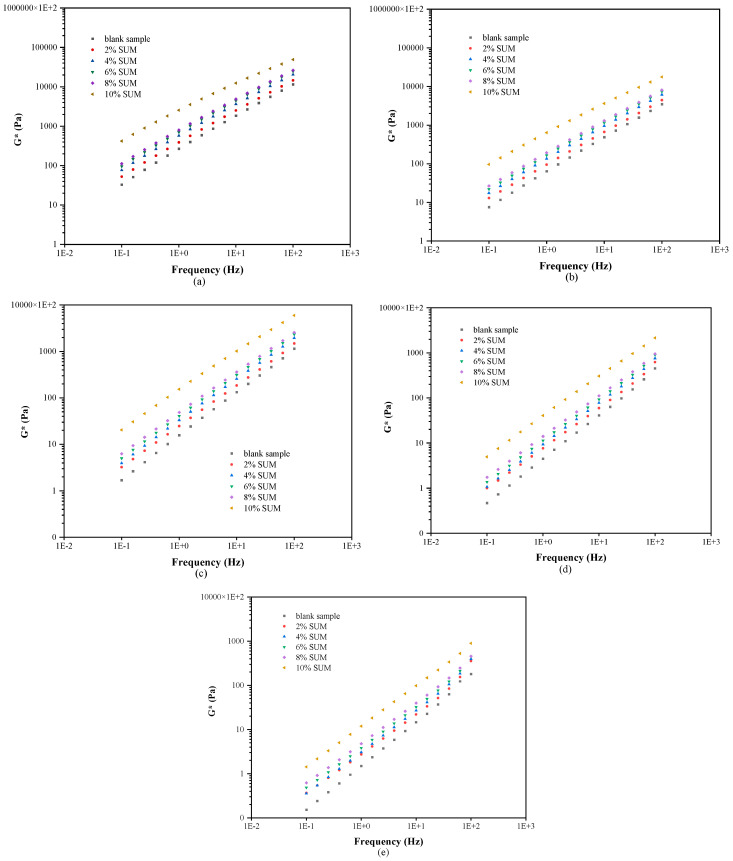
FS test outcomes corresponding to (**a**) 40, (**b**) 50, (**c**) 60, (**d**) 70, and (**e**) 80 °C.

The failure temperature—used to establish the performance grade (PG) of asphalt—corresponds to the temperature where G*/sin (δ) does not exceed 1.0 kPa prior to aging and remains under 2.2 kPa following RTFO aging. Figure 8 illustrates the failure temperatures of SUMMs with varying SUM percentages both pre- and post-RTFO aging. According to Figure 8a, elevating the SUM content leads to an increase in failure temperature, contributing to a better PG–temperature grade. The failure temperature of the unmodified sample increased progressively from 67.8 °C to 80.9 °C, as the additive concentration was raised in increments of 2%, 4%, 6%, 8%, and 10%, reaching 70.6 °C, 72.6 °C, 75.5 °C, and 77.5 °C at each corresponding concentration. Figure 9b illustrates that following RTFO aging, the failure temperatures of SUMMs subjected to RTFO aging tend to rise progressively, corresponding to the elevated SUM content. According to Figure 8b, the elevation in SUM concentration correlates with an increase in the PG-temperature grade of the unmodified sample, which progresses stepwise from PG64-xx to PG70-xx, PG76-xx, and PG82-xx at each SUM concentration examined. Therefore, it is evident that SUM contributes significantly to enhancing the rutting resistance of the asphalt binder.

### 3.2. FS Results

The influence of SUM concentration on G* across multiple frequency loadings and temperature conditions is illustrated in Figure 9. As the loading frequency increased, there was a corresponding rise in G* for all asphalt binders. The elastic properties of the asphalt binder at greater frequencies during reduced loading times provide a reasonable explanation for this phenomenon. In contrast, a rise in temperature at a constant frequency caused a reduction in the asphalt’s capacity to resist rutting. Additionally, incorporating SUMs contributed to improvements in the performance characteristics of asphalt. Across a range of loading frequencies, the unmodified exhibited the minimum G*, while SUMMs containing 10% SUM content achieved the highest G* relative to the other SUMMs. For instance, under testing conditions of 0.1 Hz frequency and 80 °C, the G* measurements corresponded to 15.2 Pa for the unmodified sample and increased progressively to 35, 37, 48.5, 62.08, and 143 Pa for samples containing 2%, 4%, 6%, 8%, and 10% SUM content, respectively. Meanwhile, under conditions of 100 Hz and 70 °C, the G* value for the unmodified specimen increased by 95% to 390%, as the SUM content was raised from 2% to 10%, specifically by 95%, 117%, 145%, 151%, and 390% for 2%, 4%, 6%, 8%, and 10% additions, respectively. The data suggest that the elastic properties of asphalt binders are superior at increased frequencies after they have undergone modification.

### 3.3. Analytical Assessment and Establishment of Models Describing Characteristics of Rut Deformation

Table 4 presents the identified responses corresponding to the factors designated via the RSM software (Design-Expert 13) using the CCD method, based on 19 experimental runs conducted in a randomized sequence. To assess the statistical significance of the prediction models, ANOVA was performed, with the corresponding outcomes tabulated in Table 4. The presence of statistical significance in the model can be evaluated using the 95% confidence level alongside the F-value. As indicated in Table 4, the model F-values for G* and G*/sin (δ) confirm their significance, with a mere 0.01% chance that values of this magnitude could be attributed to noise. At a 95% confidence level, the *p*-values obtained for all models, along with their respective variables, were found to be under 0.05, establishing their statistical significance. Equations (4) and (5) illustrate the outcomes of the models that were developed.

(4) $$\begin{array}{l} G^{*}=6.75-0.0888*F_{1}-0.0602*F_{2}+0.0336*F_{3}-0.000159*F_{1}F_{2}-0.000737*\\ F_{1}F_{3}-0.000353*F_{2}F_{3}+0.000295*F_{1}^{2}-0.000417*F_{2}^{2}+0.00895*F_{3}^{2} \end{array}$$

(5) $$\begin{array}{l} G^{*}/\sin\;(\delta )\;=6.83-0.0913*F_{1}+0.0616*F_{2}+0.0323*F_{3}+0.000139*F_{1}F_{2}-\\ 0.000887*F_{1}F_{3}-0.000295*F_{2}F_{3}+0.000321*F_{1}^{2}-0.000419*F_{2}^{2}+0.00983*F_{3}^{2} \end{array}$$

Table 5 summarizes the coefficient of determination (R^2^), its adjusted value (R^2^__adj_), and the predicted R^2^ (R^2^__pre_) as derived from the ANOVA analysis. R^2^ is utilized to gauge the goodness of fit, with values above 0.8 typically indicating a model that fits the data well [70]. When a model exhibits substantial R-squared values, it tends to reflect improved consistency between the actual and predicted responses. The data tabulated in Table 5 indicate that all values for both models surpass the threshold of 0.8. Furthermore, the elevated values of both the coefficient of determination and its adjusted counterpart confirm the statistical soundness of the models and reveal a strong correspondence between the observed and predicted responses [51].

**Figure 10:**
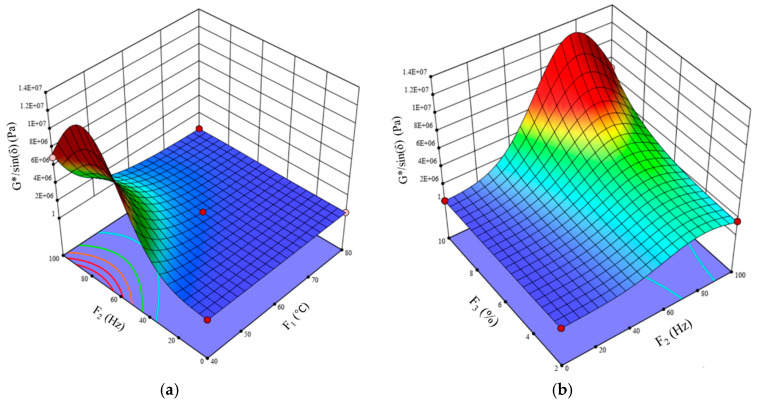
3D surface representations of G*/sin (δ) with (**a**) F_1_, F_2_ and (**b**) F_2_, F_3_.

The relationships between G*/sin (δ) and multiple independent variables are effectively represented by the three-dimensional response surface presented in Figure 10. As illustrated in Figure 10a, G*/sin (δ) exhibited higher values at elevated loading frequencies accompanied by lower temperatures. Considerable temperature variations are apparent across all frequency levels. The effect of frequency level changes on the rutting behavior of SUMMs was diminished at 80 °C relative to the more advantageous impact experienced at 40 °C. When subjected to higher temperatures, asphalt exhibits less rigidity, thereby leading to lower values of G*/sin (δ). Figure 10b illustrates that G*/sin (δ) significantly increased in response to the simultaneous rise in loading frequency and SUM content. The incorporation of SUMs enhanced the asphalt’s resistance to rutting, as demonstrated by the increased G*/sin (δ) values. In addition, SUMs considerably improved rutting performance at high loading frequencies. The data obtained from the analysis exhibit that all independent variables have a substantial influence on the rutting behavior of the binder.

### 3.4. MSCR Evaluation

**Figure 11:**
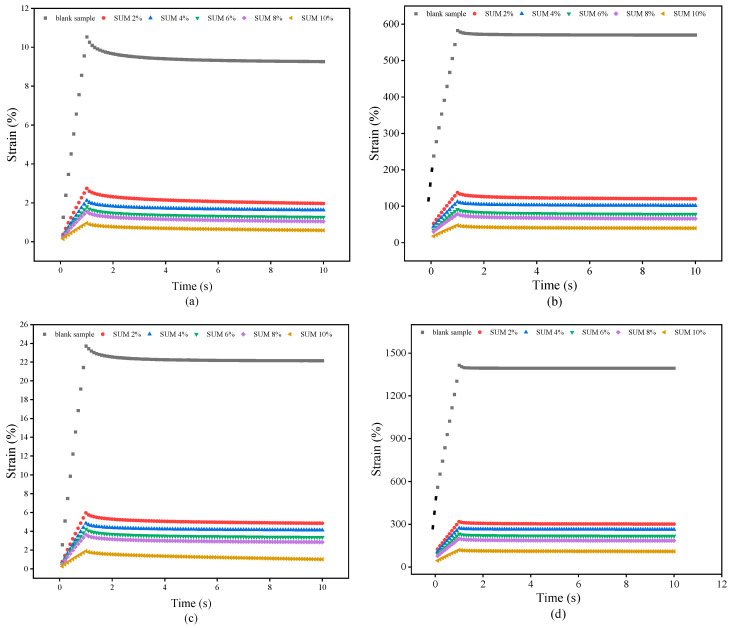
Accumulated strain (**a**) 0.1 kPa-58 °C; (**b**) 3.2 kPa-58 °C; (**c**) 0.1 kPa-64 °C; and (**d**) 3.2 kPa-64 °C.

**Figure 12:**
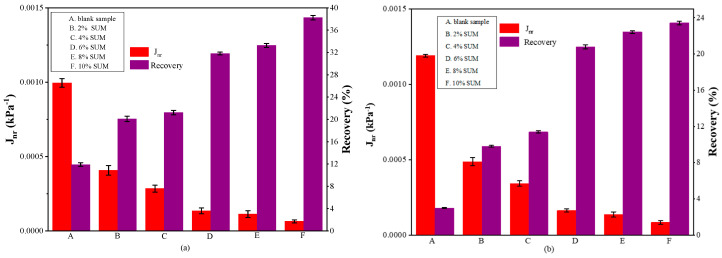
MSCR parameters (**a**) 0.1 kPa; and (**b**) 3.2 kPa at 58 °C.

**Figure 13:**
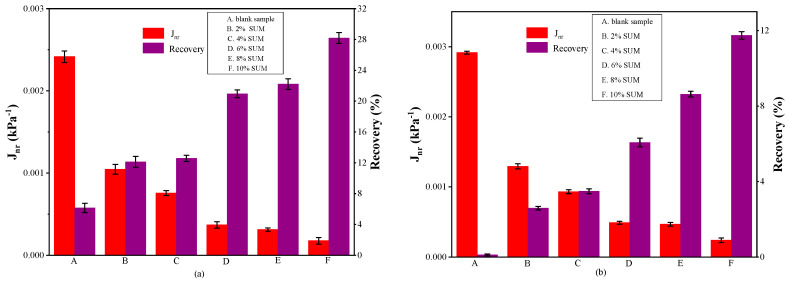
MSCR parameters (**a**) 0.1 kPa; and (**b**) 3.2 kPa at 64 °C.

The deformation behavior of SUMM and unmodified samples under different stress intensities can be elucidated through the MSCR test. Figure 11, Figure 12 and Figure 13 present the findings from the MSCR test.

The strain accumulation of the samples during the first cycle at two temperatures (58 °C and 64 °C) and under dual stress levels, each reflecting distinct traffic load scenarios on asphalt pavement, is depicted in Figure 11. The results reflect a 1 s duration of creep loading at steady stress, followed by a recovery phase of 9 s. As the loading period lengthened during the creep phase, the strain amount correspondingly escalated. The measured strain exhibited a swift recovery at the beginning of the relaxation phase, subsequently declining steadily over time. Increased cumulative strain corresponds to a higher likelihood of rut formation. Figure 11a,b clearly show that, under both stress and temperature variations, the SUMM demonstrates considerably reduced accumulated strain values relative to the unmodified binder. Figure 11a illustrates that, at 58 °C, the accumulated strain of the unmodified sample is reduced by 69.8%, 77%, 78.5%, 81.7%, and 88.1% at the end of the loading cycle, following the addition of 2%, 4%, 6%, 8%, and 10% SUM content, respectively. Findings reveal that the accumulated strain reduces in response to elevated levels of SUM content. As illustrated in Figure 11c, an increase in temperature to 64 °C at the identical stress level resulted in a similar trend, despite the unmodified sample displaying greater strain than at 58 °C. Under a stress level of 3.2 KPa, the binders reached a peak strain at the end of the creeping stage that was substantially larger in comparison with the strain measured at 0.1 KPa, a trend observed at both temperatures, as anticipated. The data shown in Figure 11 reveal improved rutting behavior under varying loading conditions, encompassing both elevated and reduced levels.

The parameters J_nr_ and R for the control and SUMM specimens with differing contents, tested at 58 °C and 64 °C under stress intensities of 0.1 and 3.2 kPa, are presented in Figure 12 and Figure 13. The data presented in Figure 12 indicate that the J_nr_ values fall progressively as the SUM content increases. At the two applied stress intensities, the J_nr_ values of SUMMs with varying dosages were consistently lower in comparison with the unmodified one. Moreover, the impact of temperature decreased gradually with rising levels of SUM content. To illustrate, at a test temperature of 64 °C and a stress level of 0.1 kPa (Figure 13a), incorporating 4% SUM resulted in a 68% reduction in the J_nr_ value relative to the blank sample. In contrast, under the same conditions, the J_nr_ associated with 8% SUMA was diminished by 87% compared to the unmodified specimen. Evidence confirmed that elevating the SUM concentration caused a decline in the impact of temperature on the binders. This observation demonstrates that increasing SUM content can lower the asphalt’s J_nr_ by decreasing its sensitivity to temperatures, thereby clearly enhancing its resistance to rutting. Measurements showed that the J_nr_ values of the unmodified sample and the SUMMs under 0.1 kPa shear stress was lower compared to those under 3.2 kPa, suggesting that J_nr_ varies with shear stress and escalates as shear stress increases. Therefore, shear stress plays a significant role in determining the asphalt’s resistance to deformation at elevated temperatures. Such findings provide a solid foundation and justification for employing modified asphalt in regions subjected to heavy loading. The rise in SUM concentration corresponds to a reduction in J_nr_ under both stress conditions, indicating that increasing the modifier content leads to a reduction in viscous deformation. Enhancing the asphalt binder’s stiffness through the inclusion of elastic constituents implies that SUMs can greatly improve durability against permanent deformation when exposed to high temperatures. Thus, greater amounts of SUM content correspond to more significant improvements in resistance to rutting.

When subjected to the same stress, asphalt binders containing SUM exhibited improved R values relative to the control sample, as exhibited in Figure 12 and Figure 13. These findings indicate that the incorporation of SUMs may improve the elastic deformation capacity of asphalt and strengthen its resistance to rutting when subjected to the same applied stress. In addition, greater SUM content leads to more significant advancements in elastic deformation and a heightened capability of the asphalt binder to resist rutting. Large measurements of R imply decreased susceptibility to rutting deformation [71]. As illustrated in Figure 12 and Figure 13, the SUMM sample with 10% SUM content exhibited the highest R value, whereas the control sample recorded the lowest. Notably, applying higher loading stress during the MSCR test on the same asphalt type resulted in a lower R value. This finding suggests that increased stress reduces elastic recovery, thereby causing greater residual strain as well as diminishing its ability to resist deformation at rising temperatures. The sensitivity of asphalt deformation to thermal condition and stresses escalates with rising loading stress levels. Based on the data, increasing the creep stress to 3.2 kPa produces higher J_nr_ values while simultaneously lowering the R values. Experiencing high levels of stress leads to the destruction of the modifier’s internal structure, resulting in poorer recovery behavior than during exposure to minimal stress of 0.1 kPa. These results suggest that elevated stress levels lead to increased permanent deformation in the binder, which exhibits limited capacity to recover.

The sensitivity of SUMMs to stress was assessed via J_nr,diff_, a metric representing the percentage difference between the J_nr_ recorded at stress levels of 3.2 and 0.1 kPa in this investigation. The J_nr,diff_ was determined based on Equation (6).

(6) $$J_{nr,diff}=\frac{J_{{nr}_{3.2}}-J_{{nr}_{0.1}}}{J_{{nr}_{3.2}}}*100$$

Differences in J_nr_ values across different proportions of SUM at 58 and 64 °C are documented in Table 6. The (AASHTO M 332) standard [71] specifies that, at high temperatures, the J_nr,diff_ parameter should not exceed 75%. Data from the table reveal that all SUMM samples, regardless of their SUM content, maintained J_nr,diff_ values below 75%, suggesting that SUMMs respond less sensitively to stress fluctuations.

The findings demonstrated that increasing the SUM content led to a reduction in J_nr_ and an increase in R, indicating that asphalt binders modified with SUM possess boosted resistance to rutting compared to blank specimens. These results are consistent with those obtained from G*/sin (δ) analyses.

The R versus J_nr_ curve represented by Equation (7), as outlined by AASHTO R 92–18 and further literature [72], constitutes a reliable means of characterizing binder elasticity. If the plotted R_3.2_ versus J_nr3.2_ data surpass the standard curve, this signals a meaningful elastic response, thereby demonstrating an improvement in the elastic characteristics of the asphalt.

(7) $$R_{3.2}=29.371*{\left(J_{nr3.2}\right)}^{-0.26}$$

**Figure 14:**
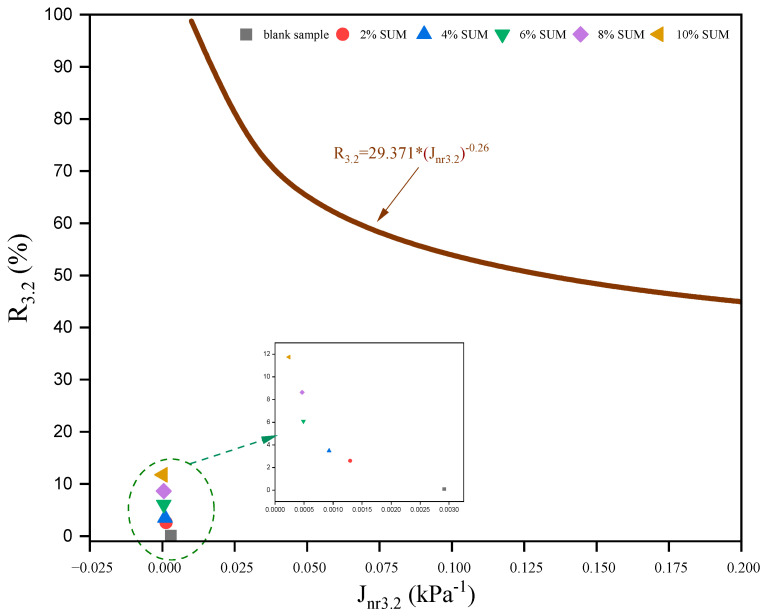
Relationship between R_3.2_ and J_nr3.2_ measurements at 64 °C.

The results at 64 °C, depicted in Figure 14, demonstrate that all R_3.2_ against J_nr3.2_ measurements for the SUMMs are situated below the baseline curve, implying reduced elastic characteristics. Nonetheless, as the proportion of SUM increased from 2% to 10%, the measured results progressively enhanced. Such a pattern implies that the inclusion of SUM strengthens the elastic performance of the asphalt binder, a conclusion supported by the MSCR test results.

### 3.5. FM Analysis

**Figure 15:**
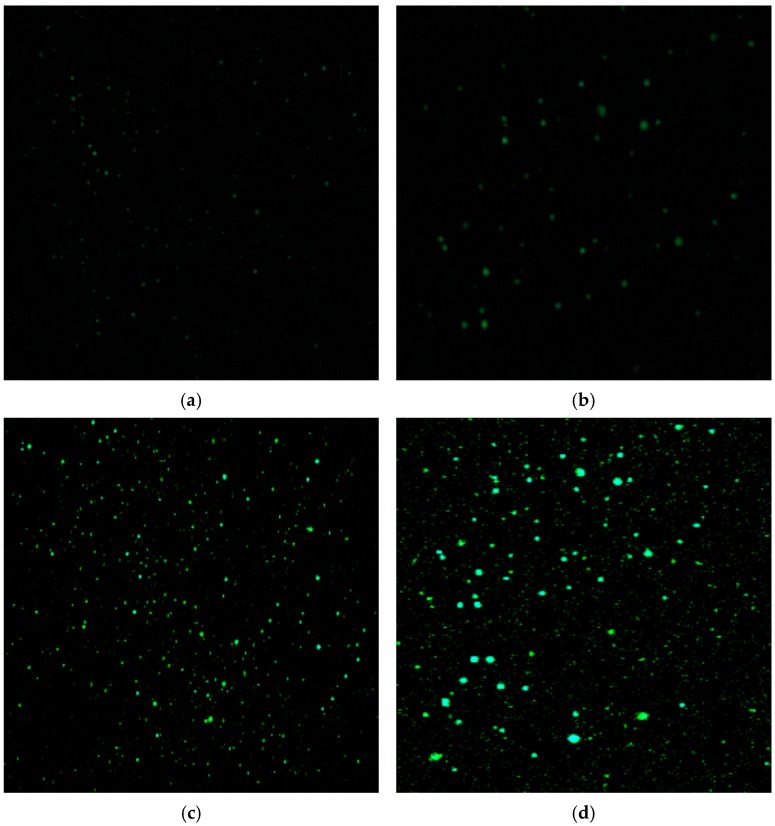
FM of SUMMs at (**a**) 4%, (**b**) 6%, (**c**) 8%, and (**d**) 10% of SUM content.

The microstructure and dispersion of the modifier were inspected using FM to provide a structural explanation for the performance enhancements observed. The FM images in Figure 15a–d show the SUM-derived polymer phase, visible as green, fluorescent particles, within the asphalt matrix.

The high-shear mixing process effectively disperses the shredded SUM into the molten asphalt. During this process, the polymer particles swell by absorbing the lighter, maltenic fractions of the bitumen, forming the distinct, dispersed polymer-rich phase visible in the FM images. The interaction mechanism is predominantly physical, a conclusion supported by the known chemical inertness of polypropylene and validated by our comprehensive rheological results. This physically formed, three-dimensional structure is the primary mechanism responsible for the performance enhancements observed, resulting from the physical restriction of asphalt molecular flow. At SUM concentrations up to 8%, the particles exhibit a uniform and homogeneous distribution with no evidence of significant agglomeration. This homogeneous dispersion is critical, as it confirms the formation of a functionally continuous, three-dimensional reinforcing structure composed of discrete polymer particles. This structure, often referred to as a polymer network in modified asphalt literature, physically reinforces the entire asphalt matrix. This reinforcing structure is the primary scientific mechanism responsible for the following rheological improvements observed throughout this study:

- Enhanced Rutting Resistance: At high temperatures, the network provides superior resistance to permanent deformation. This is quantified by the significantly improved G*/sin (δ) values seen in Figure 7, as well as the lower J_nr_ values measured in the MSCR test (Figure 12).
- Improved Elastic Recovery: The inherent elasticity of the PP network enhances the binder’s ability to recover its shape after loading. This is directly confirmed by the higher R values observed in the MSCR tests (Figure 13).

At the highest concentration of 10% SUM content, the fluorescent particles display larger average sizes and slightly reduced uniformity, suggesting the onset of localized heterogeneity or particle clustering. However, across the effective modification range, the FM analysis provides critical visual evidence that validates the link between the SUM-induced microstructure and the enhanced mechanical performance, confirming that a well-dispersed interactive polymer phase is the key mechanism of modification.

## 4. Conclusions

The academic community is widely engaged in identifying strategies that boost the functionality of asphalt surfaces, with concurrent attention given to their environmental and economic effects. Their ongoing effort has driven the utilization of unconventional waste sources, including the recycling of polymeric materials obtained from discarded personal protective equipment. Using the MSCR, temperature sweep, and frequency sweep procedures, this work sought to assess the behavior of SUMMs at elevated temperatures. Additionally, several experiments structured using Design-Expert version 13 were conducted in conjunction with RSM to analyze how SUM percentage, temperature, and loading frequency collectively affect G* and the material’s resistance to rutting, as well as the interactions among these factors. Predictive models were then proposed to accurately represent the laboratory data. In addition, the microstructural features of the modified binders were examined via FM. The key findings are presented below:

Tests involving sweeps of temperature and frequency indicated that higher SUM content enhanced the G* and G*/sin (δ) measurements while reducing δ, suggesting a positive impact of SUMs on both rutting resistance and elastic behavior. Moreover, the observed advancement in the performance grade following the incorporation of SUMs suggests that SUMMs possess strong rutting resistance, making them well suited for applications under hot climate conditions.

The outcomes of the analysis of variance demonstrated a considerable effect of loading frequency, temperature, and SUM percentage on rutting behavior. Considering the factors affecting SUMMs, temperature was identified as the dominant variable influencing the values of G* and G*/sin (δ). The RSM-generated models exhibited significant correlation coefficients, suggesting they are reliable tools for estimating SUMM’s susceptibility to rutting.

Across stress levels of 0.1 and 3.2 kPa, J_nr_ decreased and R increased following the addition of SUM, indicating improved elastic recovery as SUM enhanced the binder’s influence over the mixture’s permanent deformation.

Results obtained from the combined frequency sweep, temperature sweep, along with the MSCR analysis, demonstrated that SUM contributes beneficially to the high-temperature characteristics of asphalt binders.

The data obtained from FM demonstrated a homogeneous dispersion of SUMs within the asphalt binder matrix.

## Figures and Tables

**Table 1:** Standard properties of AH-70# asphalt binder.

Test	Result	Requirement
Penetration depth (100 g, 5 s, 25 °C), 0.1 mm	71	60–80
Penetration index PI	−0.87	−1.5–+1.0
Softening point (°C)	48	≥46
Ductility (15 °C), cm	>100	≥100
Flash point (°C)	>300	≥260
Dynamic viscosity at 60 °C (Pa s)	223	≥180

**Table 2:** Physical characteristics of SUMs.

Property	Standard	Value
Rupture force (N)	ASTM-D638-14 [54]	19.38
Tensile strength (MPa)	ASTM-D638-14 [54]	3.45
Water absorption 24 h (%)	ASTM-D570-98 [55]	8.2
Melting point (°C)	ASTM-D7138-16 [56]	155
Specific gravity	ASTM-D792-20 [57]	0.91

**Table 3:** Data corresponding to experimental design.

Run	Factors			Responses	
	F_1_: Temperature (°C)	F_2_: Frequency (Hz)	F_3_: SUM (%)	G*/sin (δ) (KPa)	G* (KPa)
1	60	50.05	6	126	124
2	60	100	6	240	234
3	60	50.05	6	129	126
4	80	0.1	2	0.038	0.037
5	40	0.1	2	5.40	5.28
6	60	50.05	6	122	118
7	80	100	2	35.4	35.4
8	60	50.05	2	77.4	75.0
9	80	0.1	10	0.147	0.146
10	60	50.05	10	399	364
11	40	100	10	7050	5180
12	40	50.05	6	1670	1500
13	40	0.1	10	46.9	44.6
14	80	100	10	93.0	90.8
15	60	50.05	6	133	130
16	80	50.05	6	16.2	16.2
17	60	0.1	6	0.507	0.503
18	60	50.05	6	128	126
19	40	100	2	1630	1470

**Table 4:** Variance analysis conducted for response variables.

Responses	Source	SS	df	MS	F-Value	*p*-Value
G*/sin (δ)	Model	32.8	9	3.64	3990	<0.001
	F_1_	10.4	1	10.4	11,400	<0.001
	F_2_	17.2	1	17.2	18,800	<0.001
	F_3_	1.09	1	1.09	1190	<0.001
	F_1_F_2_	0.155	1	0.155	170	<0.001
	F_1_F_3_	0.0403	1	0.0403	44.2	<0.001
	F_2_F_3_	0.0277	1	0.0277	30.4	0.0004
	F_1_^2^	0.0452	1	0.0452	49.6	<0.001
	F_2_^2^	2.98	1	2.98	3270	<0.001
	F_3_^2^	0.0676	1	0.0676	74.2	<0.001
G*	Model	31.9	9	3.54	2490	<0.001
	F_1_	9.96	1	9.96	6990	<0.001
	F_2_	16.8	1	16.8	11,800	<0.001
	F_3_	1.00	1	1.00	705	<0.001
	F_1_F_2_	0.201	1	0.201	141	<0.001
	F_1_F_3_	0.0278	1	0.0278	19.5	0.0017
	F_2_F_3_	0.0397	1	0.0397	27.9	0.0005
	F_1_^2^	0.0380	1	0.0380	26.7	0.0006
	F_2_^2^	2.95	1	2.95	2070	<0.001
	F_3_^2^	0.0561	1	0.0561	39.4	0.0001

**Table 5:** Metrics assessing model suitability.

Response	R^2^	R^2^__pre_	R^2^__adj_
G*/sin (δ)	0.9997	0.9972	0.9995
G*	0.9996	0.9950	0.9992

**Table 6:** Percentage values of J_nr,diff_.

SUMM (%)	J_nr,diff_ Values (%)	
	58 °C	64 °C
0	19.26	21.90
2	19.50	23.78
4	20.0	23.06
6	20.74	26.22
8	20.20	28.17
10	30.29	35.66

## Data Availability

The original contributions presented in this study are included in the article. Further inquiries can be directed to the corresponding authors.
